# GPNMB plays a protective role against obesity-related metabolic disorders by reducing macrophage inflammatory capacity

**DOI:** 10.1016/j.jbc.2021.101232

**Published:** 2021-09-25

**Authors:** Adam Prabata, Koji Ikeda, Elda Putri Rahardini, Ken-Ichi Hirata, Noriaki Emoto

**Affiliations:** 1 Laboratory of Clinical Pharmaceutical Science, Kobe Pharmaceutical University, Kobe, Japan; 2 Division of Cardiovascular Medicine, Department of Internal Medicine, Kobe University Graduate School of Medicine, Kobe, Japan; 3 Department of Epidemiology for Longevity and Regional Health, Kyoto Prefectural University of Medicine, Kyoto, Japan

**Keywords:** obesity, macrophage, adipose tissue, inflammation, metabolic disease, adipocyte, GPNMB (glycoprotein nonmetastatic melanoma protein B), ECD, extracellular domain, FBS, fetal bovine serum, GPNMB, glycoprotein nonmetastatic melanoma protein B, HFD, high-fat diet, SVF, stromal vascular fraction, TEPM, thioglycolate-elicited peritoneal macrophage, WAT, white adipose tissue, WT, wild-type

## Abstract

Obesity is a global health problem that is often related to cardiovascular and metabolic diseases. Chronic low-grade inflammation in white adipose tissue (WAT) is a hallmark of obesity. Previously, during a search for differentially expressed genes in WAT of obese mice, we identified glycoprotein nonmetastatic melanoma protein B (*GPNMB*), of which expression was robustly induced in pathologically expanded WAT. Here, we investigated the role of GPNMB in obesity-related metabolic disorders utilizing GPNMB-deficient mice. When fed a high-fat diet (HFD), GPNMB-deficient mice showed body weight and adiposity similar to those of wild-type (WT) mice. Nonetheless, insulin and glucose tolerance tests revealed significant obesity-related metabolic disorders in GPNMB-KO mice compared with WT mice fed with HFD. Chronic WAT inflammation was remarkably worsened in HFD-fed GPNMB-KO mice, accompanied by a striking increase in crown-like structures, typical hallmarks for diseased WAT. Macrophages isolated from GPNMB-KO mice were observed to produce more inflammatory cytokines than those of WT mice, a difference abolished by supplementation with recombinant soluble GPNMB extracellular domain. We demonstrated that GPNMB reduced the inflammatory capacity of macrophages by inhibiting NF-κB signaling largely through binding to CD44. Finally, we showed that macrophage depletion by addition of clodronate liposomes abolished the worsened WAT inflammation and abrogated the exacerbation of metabolic disorders in GPNMB-deficient mice fed on HFD. Our data reveal that GPNMB negatively regulates macrophage inflammatory capacities and ameliorates the WAT inflammation in obesity; therefore we conclude that GPNMB is a promising therapeutic target for the treatment of metabolic disorders associated with obesity.

Obesity is increasing worldwide; its prevalence has nearly tripled between 1975 and 2016, and over 650 million adults were obese in 2016 according to a report of World Health Organization. Obesity is closely and causally associated with cardiovascular disease, diabetes, and some cancers, and thus it has been a global health issue in the world (1). In obesity, white adipose tissue (WAT) expands due to increase and hypertrophy of adipocytes. During WAT expansion, adipocytes are exposed to various stresses such as metabolic, oxidative, and endoplasmic reticulum stress as well as hypoxia due to imbalanced AT angiogenesis, leading to low-grade chronic inflammation in the WAT (2, 3, 4, 5). Macrophages aggressively infiltrate into the WAT during obesity and accelerate the chronic inflammation that is causally implicated in insulin resistance in the WAT and in systemic as well (6, 7). Therefore, ameliorating the WAT inflammation, especially by modulating the macrophage functions, is a promising therapeutic strategy for the treatment of metabolic disorders associated with obesity.

To identify previously unknown mechanisms underlying the WAT chronic inflammation in obesity, we searched for genes that are differentially regulated in the WAT of obese mice comparing to that of lean mice. Accordingly, we found that glycoprotein nonmetastatic melanoma protein B (GPNMB) is highly expressed in the WAT of obese mice. GPNMB is a type-I transmembrane protein, the soluble form of which is secreted by a disintegrin and metalloprotease domain 10 (AMDM10)-mediated cleavage (8). GPNMB has been originally identified from a nonmetastatic melanoma cell line and xenograft. Subsequent studies revealed that GPNMB is expressed not only in cancer cells but also in many normal cells including macrophage, dendritic cell, osteoblast, microglia, and neuron, and it has been associated with various pathological conditions such as colitis, nonalcoholic steatohepatitis, amyotrophic lateral sclerosis, glaucoma, and neuroinflammation in addition to cancer progression, while its causal relevance in these diseases remains unclear (8, 9, 10). The soluble GPNMB extracellular fragment has been reported to bind to and/or interact with various receptors/effectors such as Na^+^-K^+^-ATPase, CD44, epidermal growth factor receptor, integrins, heparin, and Syndecan-4 (10). Soluble GPNMB modulates various signal pathways including ERK/MERK, Akt/PI3K, and NF-κB pathways to mediate its biological functions (10). GPNMB has been closely associated with inflammation and immune response, and many reports suggested its anti-inflammatory role, while some studies suggested its proinflammatory role. Therefore, its role in inflammation has not been fully understood.

Recently, it has been reported that GPNMB expressed in adipocytes ameliorated the fat accumulation and fibrosis of the liver in diet-induced obesity model without affecting obesity and adiposity (11). On the other hand, there was a report that GPNMB secreted from liver promotes lipogenesis in WAT and aggravates diet-induced obesity and insulin resistance (12). Therefore, a role of GPNMB in obesity and its-associated metabolic disorders remains controversial. Here, we explored a role of GPNMB in obesity using GPNMB-deficient mice and revealed its protective role in obesity-related metabolic disorders by reducing macrophage inflammatory capacities.

## Results

### GPNMB is highly expressed in the WAT of obese mice

**Figure 1:**
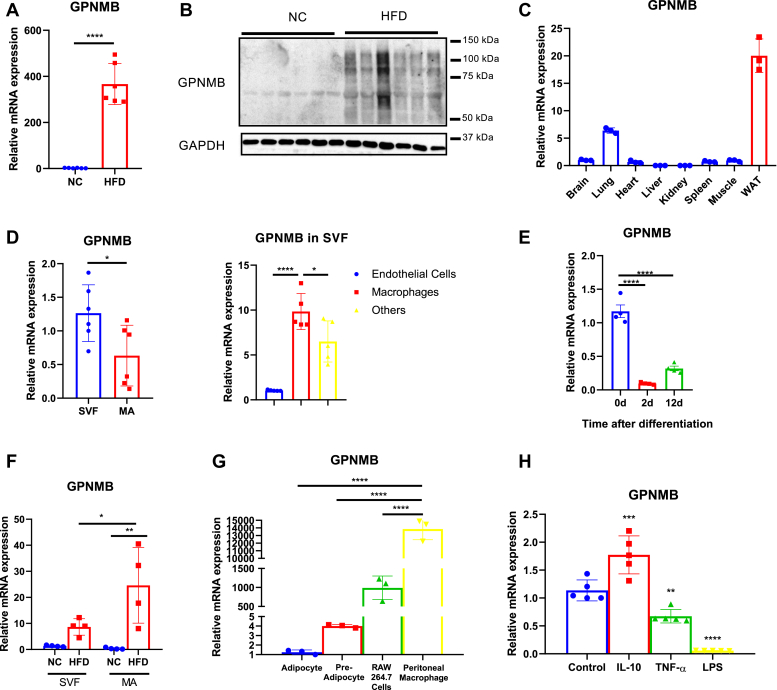
**GPNMB expression is robustly increased in the WAT of obese mice.***A*, quantitative PCR for GPNMB in the WAT isolated from either lean mice fed normal chow (NC) or obese mice fed an high-fat diet (HFD) for 12 weeks (n = 6 each). *B*, immunoblotting for GPNMB in the WAT isolated from lean mice fed a NC or obese mice fed an HFD (n = 6 each). *C*, quantitative PCR for GPNMB in various tissues of lean mice (n = 3 each). *D*, quantitative PCR for GPNMB in stromal vascular fraction (SVF) and mature adipocyte (MA) of the WAT isolated from lean mice (n = 6 each; *left*). Cells in SVF were separated into endothelial cells, macrophages, and others by using magnetic-activated cell sorting. GPNMB expressions in these cells were analyzed (n = 5 each; *right*). *E*, quantitative PCR for GPNMB in 3T3-L1 preadipocytes at the indicated time after induction of adipocyte differentiation (n = 4 each). *F*, quantitative PCR for GPNMB in SVF and MA of the WAT isolated from lean mice fed NC or obese mice fed an HFD (n = 4 each). *G*, quantitative PCR for GPNMB in 3T3L1-derived adipocytes, 3T3L1 preadipocytes, RAW264.7 macrophages, and mouse resident peritoneal macrophages (n = 3 each). *H*, mouse resident peritoneal macrophages were treated either vehicle (control), IL-10 (10 ng/ml), TNF-α (10 ng/ml) or LPS (10 ng/ml) for 24 h, and then GPNMB expression was analyzed (n = 5 each). Data represent mean ± SDM. ∗*p* < 0.05, ∗∗*p* < 0.01, ∗∗∗*p* < 0.001 and ∗∗∗∗*p* < 0.0001. Two-tailed Student’s *t* test was used for the analysis of the differences between the groups (*A* and *D left*), while one-way ANOVA with Tukey’s post hoc test for multiple comparisons was used for the analysis of the differences between each group (*D right*, *E*–*H*).

Because the WAT inflammation is closely associated with the adipocyte and immune cell interaction, we have performed signal sequence trap, a cloning strategy for secreted and type-I membrane proteins, using cDNA libraries prepared from the WAT of obese mice. One of genes identified encodes GPNMB, and we found that its expression in the WAT was substantially enhanced during obesity (Fig. 1, *A* and *B*). GPNMB showed relatively high expression in the WAT comparing to other tissues (Fig. 1*C*), and its expression was predominant in the stromal vascular fraction (SVF) rather than in mature adipocytes in the WAT of lean mice (Fig. 1*D*). We further dissected GPNMB expression by isolating endothelial cells and macrophages from the SVF, and found that adipose tissue macrophages highly express GPNMB (Fig. 1*D*). Because the SVF also contains preadipocytes, we analyzed expressional changes of GPNMB during adipogenesis. GPNMB expression in 3T3-L1 preadipocytes was substantially reduced at the early stage of adipogenesis (2 days after differentiation), and its expression levels remained low in mature adipocytes (12 days after differentiation) (Fig. 1*E*). In obese condition, GPNMB expression was substantially enhanced in mature adipocytes to the levels beyond that in the SVF, while its expression was also increased in the SVF (Fig. 1*F*). Analysis of GPNMB expression levels in various types of cells revealed the predominant GPNMB expression in macrophages comparing to that in preadipocytes and mature adipocytes (Fig. 1*G*). Of note, mouse resident peritoneal macrophages showed remarkably high GPNMB expression levels (Fig. 1*G*). Furthermore, inflammatory stimuli by TNF-α and LPS reduced GPNMB expression in resident peritoneal macrophages, while anti-inflammatory stimulation by IL-10 increased it (Fig. 1*H*). These data strongly suggest a crucial role of GPNMB in macrophage biology. Also, the robust increase of GPNMB in mature adipocytes during obesity suggests an involvement of GPNMB in obesity-related adipocyte dysfunction, while the high GPNMB expression in the SVF might reflects the infiltration of macrophages in the WAT during obesity.

### Genetic loss of GPNMB exacerbates metabolic disorders associated with obesity

**Figure 2:**
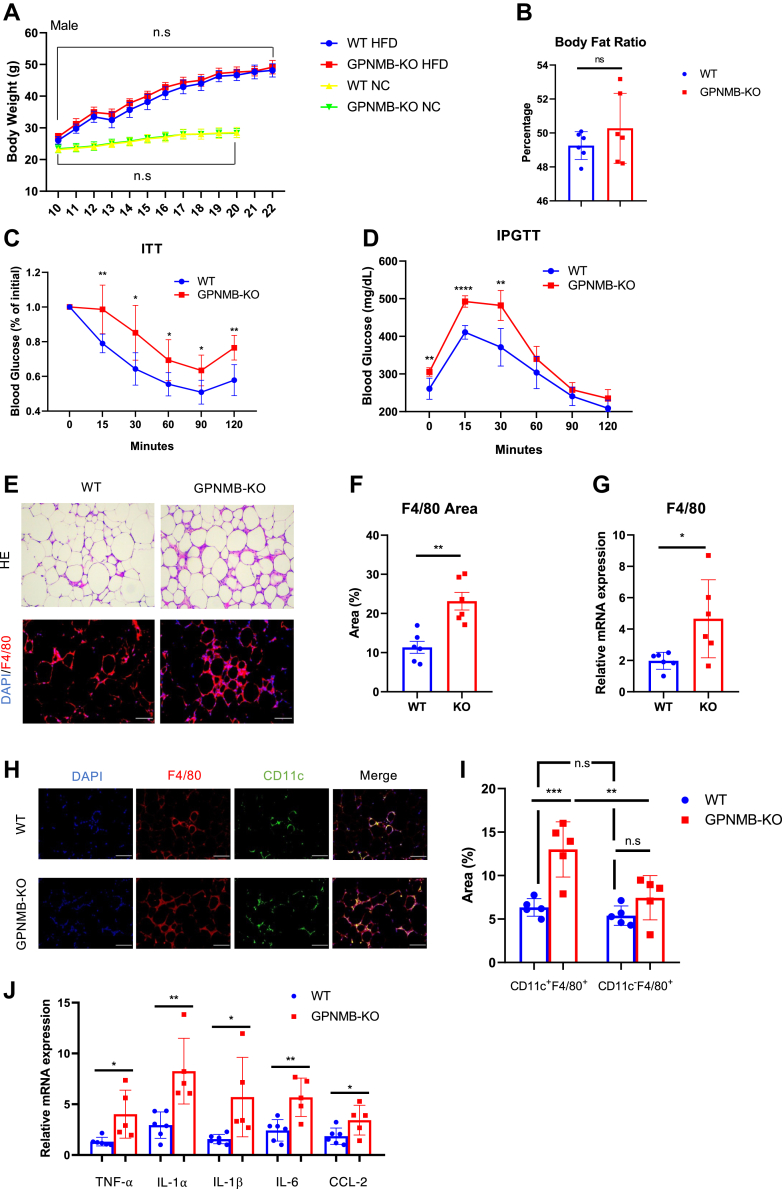
**Loss of GPNMB exacerbates obesity-related metabolic disorders.***A*, body weight of male WT and GPNMB-KO mice fed with either normal chow (NC) or high-fat diet (HFD) at the indicated ages of weeks (n = 6 each for NC group; n = 11 for WT-HFD; n = 6 for KO-HFD). The difference in body weight was not significant (n.s.) between WT and GPNMB-KO mice fed with the same diet. *B*, body fat ratio (% of fat mass in total body mass) was assessed by CT analysis in WT and GPNMB-KO mice fed an HFD for 12 weeks (n = 6 each). *C*, insulin tolerance test (ITT), in which insulin-mediated blood sugar reduction is assessed, in male WT and GPNMB-KO mice fed an HFD for 12 weeks (n = 7 for WT; n = 5 for KO). *D*, intraperitoneal glucose tolerance test (IPGTT), in which blood sugar increase after glucose administration is assessed, in male WT and GPNMB-KO mice fed an HFD for 12 weeks (n = 7 for WT; n = 5 for KO). *E*, representative images of hematoxylin and eosin (H-E) staining (*upper*) and immunohistochemistry for macrophage marker F4/80 (*lower*) in the WAT isolated from male WT and GPNMB-KO mice fed an HFD for 14 weeks. Bars: 100 μm. *F*, macrophage infiltration in the WAT was assessed by quantification of F4/80-positive area in the immunohistochemistry shown in (*E*) (n = 6 each). *G*, quantitative PCR for F4/80 in the WAT isolated from WT and GPNMB-KO mice fed an HFD for 14 weeks (n = 6 each). *H*, representative images of immunohistochemistry for F4/80 and CD11c in the WAT isolated from male WT and GPNMB-KO mice fed an HFD for 14 weeks. Bars: 50 μm. *I*, quantitative analysis of CD11c- and F4/80-double positive or CD11c-negative and F4/80-positive areas in the immunohistochemistry shown in H (n = 5 each). *J*, quantitative PCR for TNF-α, IL-1α, IL-1β, IL-6, and CCL-2 in the WAT isolated from male WT and GPNMB-KO mice fed an HFD for 14 weeks (n = 6 for WT; n = 5 for KO). Data represent mean ± SDM. ∗*p* < 0.05, ∗∗*p* < 0.01, ∗∗∗*p* < 0.001, and ∗∗∗∗*p* < 0.0001. Two-tailed Student’s *t* test was used for the analysis of the differences between groups (*A*–*D*, *F*, *G*, and *J*), while one-way ANOVA with Tukey’s post hoc test for multiple comparisons was used for the analysis of the differences between each group (*I*). n.s., not significant.

To elucidate a role of GPNMB in obesity, we generated mice with target deletion of GPNMB (GPNMB-KO) and analyzed their metabolic phenotype. When fed a normal chow, GPNMB-KO mice showed body weight, insulin sensitivity, and glucose tolerance similar to those in wild-type (WT) mice in both male (Fig. 2*A* and Fig. S1*B*) and female (Fig. S1, *A* and *C*). When fed a high-fat diet (HFD), GPNMB-KO mice showed weight gain similar to WT mice (male: Fig. 2*A* and female: Fig. S1*A*). Of note, male GPNMB-KO mice showed exacerbated metabolic disorders associated with obesity, despite similar adiposity (Fig. 2, *B*–*D*). Histological analysis revealed remarkably increased crown-like structures and enhanced macrophage infiltration in the WAT of male GPNMB-KO mice comparing to those in WT mice fed with HFD (Fig. 2, *E–G*). Adipose tissue macrophages (ATMs) have been classified into two major subtypes, *e.g.*, tissue resident and recruited ATMs (13, 14, 15). In obese condition, many recruited macrophages, which are classically activated proinflammatory macrophages, infiltrate into the WAT and are typically found in crown-like structures (13, 14, 16). CD11c has been a useful marker to distinguish recruited ATMs from resident ATMs, and therefore we detected recruited ATMs in the WAT of obese mice using immunohistochemistry for CD11c. The majority of ATMs in the WAT of obese mice were CD11c-positive recruited macrophages in both WT and GPNMB-KO mice (Fig. 2, *H* and *I*). Notably, obese GPNMB-KO mice showed significant increase of recruited ATMs, while resident ATMs showed minimal changes between WT and GPNMB-KO mice fed with HFD (Fig. 2*I*). Consistently, chronic inflammation in the WAT was deteriorated in GPNMB-KO mice comparing to that in WT mice fed an HFD (Fig. 2*J*). In addition, hepatosteatosis was also deteriorated in association with higher inflammatory cytokines expression in the liver of GPNMB-KO mice comparing to that in WT mice fed with HFD (Fig. S2, *A* and *B*). Serum triglycerides and free fatty acid levels were similar between WT and GPNMB-KO mice fed with HFD, while serum cholesterol levels were higher in GPNMB-KO mice than in WT mice (Fig. S2*C*). These data revealed a protective role of GPNMB against obesity-related metabolic disorders without affecting obesity and/or adiposity.

Somewhat surprisingly, female GPNMB-KO mice showed similar insulin sensitivity and glucose tolerance even after the HFD feeding (Fig. S1*D*). These data may suggest a gender-specific role of GPNMB; however, because female mice are relatively resistant against diet-induced obesity (Fig. 2*A* and Fig. S1*A*), careful consideration is required, and these results may suggest that the protective role of GPNMB against obesity-related metabolic disorders might be significant in considerably obese condition but not in mild obese condition.

### GPNMB regulates macrophage inflammatory capacity

**Figure 3:**
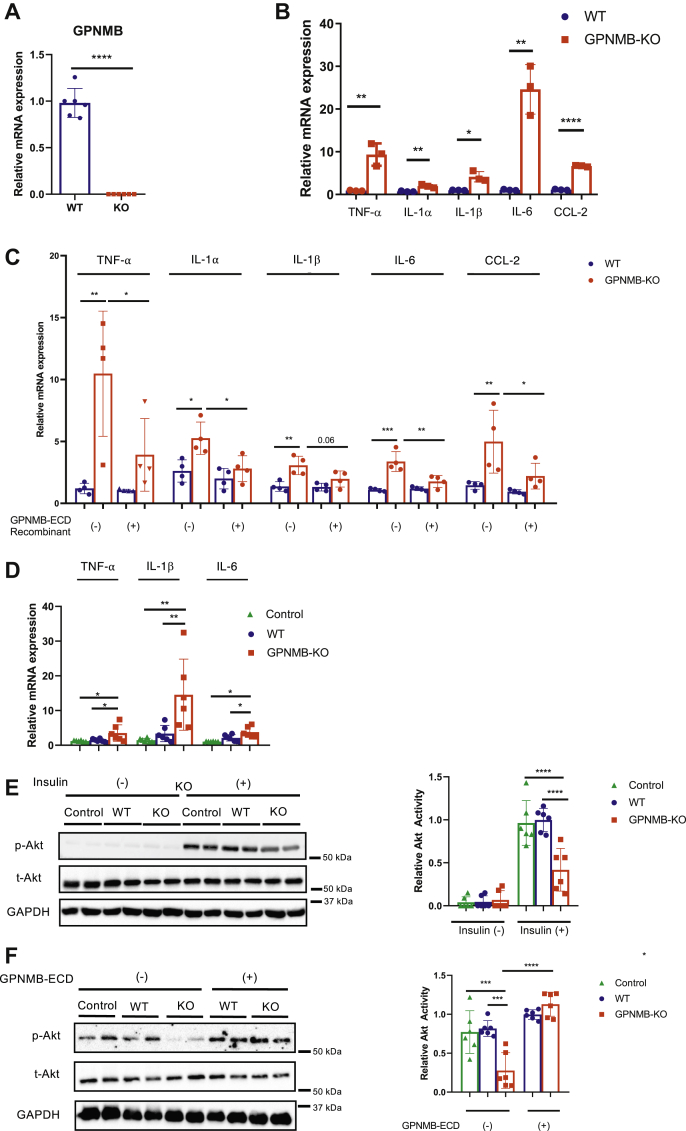
**GPNMB negatively regulates the inflammatory capacity in macrophages.***A*, quantitative PCR for GPNMB in TEPMs isolated from female WT and GPNMB-KO mice (n = 6 each). *B*, quantitative PCR for TNF-α, IL-1α IL-1β, IL-6, and CCL2 in TEPMs isolated from female WT and GPNMB-KO mice (n = 3 each). *C*, quantitative PCR for TNF-α, IL-1α IL-1β, IL-6, and CCL2 in TEPMs with or without supplementation of recombinant GPNMB extracellular domain (GPNMB-ECD) (n = 4 each). *D* and *E*, conditioned medium (CM) collected from TEPMs isolated from female WT and GPNMB-KO mice was given to 3T3-L1 adipocytes. Medium that was incubated without cells was used as control. Subsequently, inflammatory cytokines (TNF-α, IL-1β, and IL-6) expression was analyzed in adipocytes. (*D*, n = 6 each). Adipocytes were stimulated with insulin, and insulin signaling was analyzed by immunoblotting. Insulin-mediated Akt activation was quantified (*E*, n = 6 each). *F*, CM collected from TEPMs isolated from female WT and GPNMB-KO mice was given to 3T3-L1 adipocytes. Before preparation of the CM, TEPMs were treated with and without GPNMB-ECD. Medium incubated without cells was used as control. Adipocytes were stimulated with insulin, and insulin signaling was analyzed by immunoblotting. Insulin-mediated Akt activation was quantified (n = 6 each). Data represent mean ± SDM. ∗*p* < 0.05, ∗∗*p* < 0.01, ∗∗∗*p* < 0.001, and ∗∗∗∗*p* < 0.0001. Two-tailed Student’s *t* test was used for the analysis of the differences between groups (*A* and *B*), while one-way ANOVA with Tukey’s post hoc test for multiple comparisons was used for the analysis of the differences between each group (*C*–*F*).

Considering the augmented macrophage infiltration and deteriorated inflammation in the WAT of obese GPNMB-KO mice, we presumed that GPNMB negatively regulates macrophage inflammatory capacities. In fact, it has been reported that GPNMB is a feedback regulator of proinflammatory responses in macrophages (17). We isolated thioglycolate-elicited peritoneal macrophages (TEPMs) from WT and GPNMB-KO mice (Fig. 3*A*) and found that inflammatory cytokines expression was significantly enhanced in TEPMs of GPMNB-KO mice comparing to that of WT mice (Fig. 3*B*). Because secreted form of GPNMB extracellular domain (ECD) is known to be biologically active, we treated these TEPMs with recombinant soluble GPNMB-ECD and assessed the inflammatory cytokines expression. The serum concentration of GPNMB in mice has been reported to be ∼3 ng/ml (11), and we found that 1 ng/ml recombinant GPMNB-ECD showed GPNMB protein levels comparable to those in the WAT of obese mice (∼10 mg/ml total protein) (Fig. S3*A*). Therefore, we used recombinant GPNMB-ECD at 1 ng/ml for the following experiments.

Supplementation of recombinant GPNMB-ECD significantly reduced inflammatory cytokines expression in TEPMs of GPNMB-KO mice to the levels similar to that in TEPMs of WT mice (Fig. 3*C*). Furthermore, siRNA-mediated silencing for GPNMB caused significantly enhanced inflammatory activation by LPS in RAW264.7 macrophages, which was abrogated by recombinant GPNMB-ECD supplementation (Fig. S3, *B* and *C*). These data strongly suggest that GPNMB negatively regulates inflammatory capacities in macrophages largely through its soluble secreted form of the ECD in an autocrine manner.

We then examined the interaction between macrophages and adipocytes with regard to GPNMB. We overexpressed GPNMB in 3T3-L1 adipocytes using lentivirus (Fig. S4, *A* and *B*) and collected conditioned medium (CM) enriched with soluble secreted GPNMB-ECD. The CM derived from GPNMB-overexpressing adipocytes inhibited the LPS-mediate inflammatory activation in RAW264.7 macrophages (Fig. S4*C*). We next treated 3T3-L1 adipocytes with CM prepared from TEPMs isolated from WT or GPNMB-KO mice. Treatment with the CM prepared from GPNMB-KO TEPMs enhanced inflammatory cytokines expression (Fig. 3*D*) and impaired insulin signaling in 3T3-L1 adipocytes (Fig. 3*E*). Notably, supplementation of recombinant GPNMB-ECD in GPNMB-KO TEPMs abolished their detrimental effects on insulin signaling in adipocytes (Fig. 3*F*). These data strongly suggest a protective role of GPNMB in adipocyte functions through the adipocyte–macrophage interaction.

In contrast to the significant role of GPNMB in macrophages, lentivirus-mediated transfection of GPNMB affected neither inflammatory cytokines expressions (Fig. S5*A*), adipocyte maturation markers expressions (Fig. S5*B*), nor insulin signaling (Fig. S5*C*) in 3T3-L1 adipocytes. Also, lipid accumulation in adipocytes was not affected by GPNMB overexpression (Fig. S5, *D* and *E*). These data suggest a minimal role of GPNMB in adipocyte biology, and therefore GPNMB expressed in mature adipocytes in the WAT of obese mice presumably acts on ATMs in a paracrine manner.

### GPNMB negatively regulates macrophage inflammatory capacities *via* CD44

**Figure 4:**
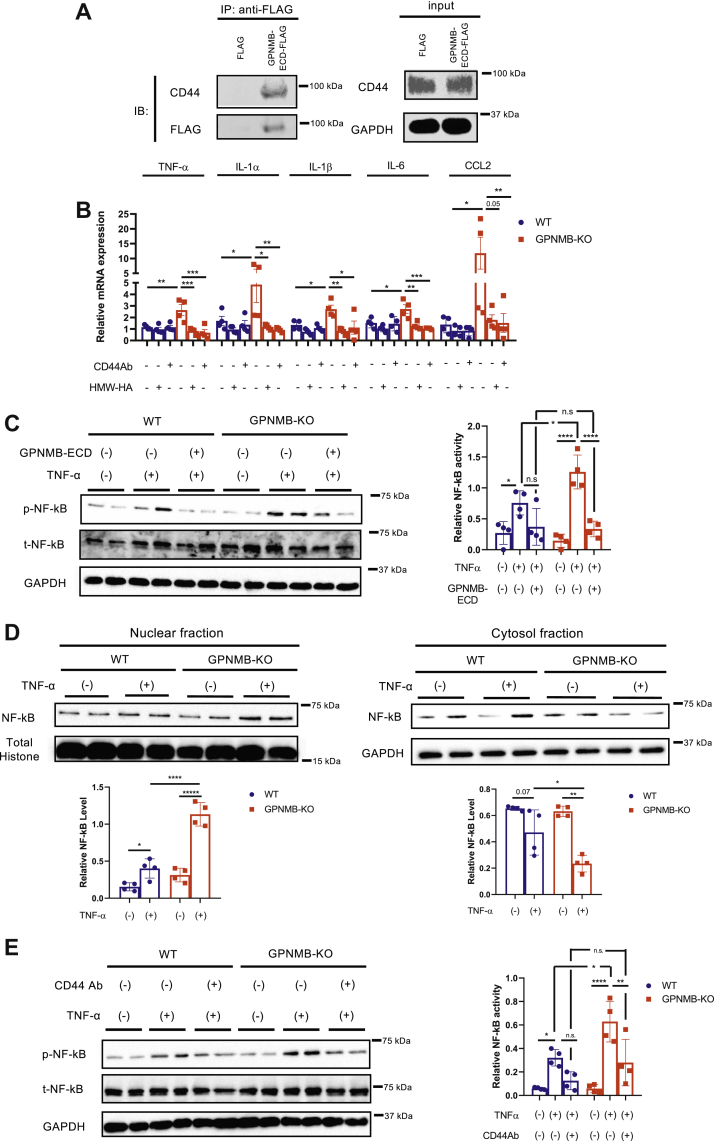
**GPNMB inhibits the activation and nuclear translocation of NF-κB through binding to CD44 in macrophages.***A*, FLAG-tagged GPNMB-ECD or FLAG-tag was transfected into CHO cells, and the CM was collected. RAW264.7 cells were incubated with the CM for 24 h, followed by protein extraction. FLAG-tagged GPNMB-ECD was immunoprecipitated, and coprecipitation of CD44 was detected by immunoblotting. *B*, quantitative PCR for TNF-α, IL-1α IL-1β, IL-6, and CCL2 in TEPMs isolated from female WT and GPNMB-KO mice in the presence or absence of CD44 antibody or high-molecular-weight hyaluronan (HMW-HA) (n = 4 each). *C*, TEPMs isolated from female WT and GPNMB-KO mice were treated with TNF-α, and NF-κB activation was assessed by immunoblotting for phospho-NF-κB p65 (n = 4 each). Some cells were treated with recombinant GPNMB-ECD. *D*, immunoblotting for NF-κB p65 in TEPMs treated with vehicle or TNF-α. Proteins were fractionated into nuclear and cytosolic fractions (n = 4 each). *E*, TEPMs isolated from female WT and GPNMB-KO mice were treated with TNF-α in the presence or absence of CD44 antibody, and NF-κB activation was assessed by immunoblotting for phospho-NF-κB (n = 4 each). Data represent mean ± SDM. ∗*p* < 0.05, ∗∗*p* < 0.01, ∗∗∗*p* < 0.001, and ∗∗∗∗*p* < 0.0001. One-way ANOVA with Tukey’s post hoc test for multiple comparisons was used for the analysis of the differences between each group (*B*–*E*). n.s., not significant.

It has been reported that GPNMB modulates cellular functions through an interaction with CD44 (18, 19, 20), and CD44 is crucially involved in macrophage inflammatory responses (21, 22, 23). Moreover, we found that CD44 was highly expressed in macrophages (Fig. S6*A*), and its expression in the WAT was substantially enhanced in obese mice in contrast to that in the liver and skeletal muscle (Fig. S6, *B* and *C*). We therefore examined whether GPNMB regulates macrophage inflammatory capacities *via* CD44. We first identified that GPNMB-ECD binds to CD44 expressed in RAW264.7 macrophages (Fig. 4*A*). Of note, inhibition of CD44 using CD44 antibody abolished the enhanced inflammatory capacity in PMs isolated from GPNMB-KO mice (Fig. 4*B*). Furthermore, high-molecular-weight hyaluronan, which is an inhibitory ligand for CD44 (22, 24, 25, 26), also abrogated the enhanced inflammatory capacity in PMs isolated from GPNMB-KO mice (Fig. 4*B*). These data strongly suggest a critical role of CD44 in the anti-inflammatory role of GPNMB.

Because NF-κB signaling plays a critical role in the inflammatory responses, we investigated the effects of GPNMB on NF-κB activation in macrophages. Activation and nuclear translocation of NF-κB in response to TNF-α was enhanced in TEPMs isolated from GPNMB-KO mice assessed by enhanced phosphorylation of NF-κB p65 and increase of NF-κB p65 in the nuclear fraction of proteins (Fig. 4, *C* and *D*). Supplementation of recombinant GPNMB-ECD canceled the enhanced NF-κB activation in GPNMB-KO TEPMs, further supporting a critical role of GPNMB-ECD in the NF-κB signaling pathway in macrophages (Fig. 4*C*). CD44 inhibition reduced the NF-κB activation in response to TNF-α in TEPMs and abolished the enhanced NF-κB activation in GPNMB-KO TEPMs (Fig. 4*E*). These data strongly suggest that GPNMB negatively regulates macrophage inflammatory capacities by blocking the CD44-mediated accentuation of NF-κB signaling.

### Macrophage dysfunction plays an essential role in the exacerbated obesity-related metabolic disorders in GPNMB-KO mice

**Figure 5:**
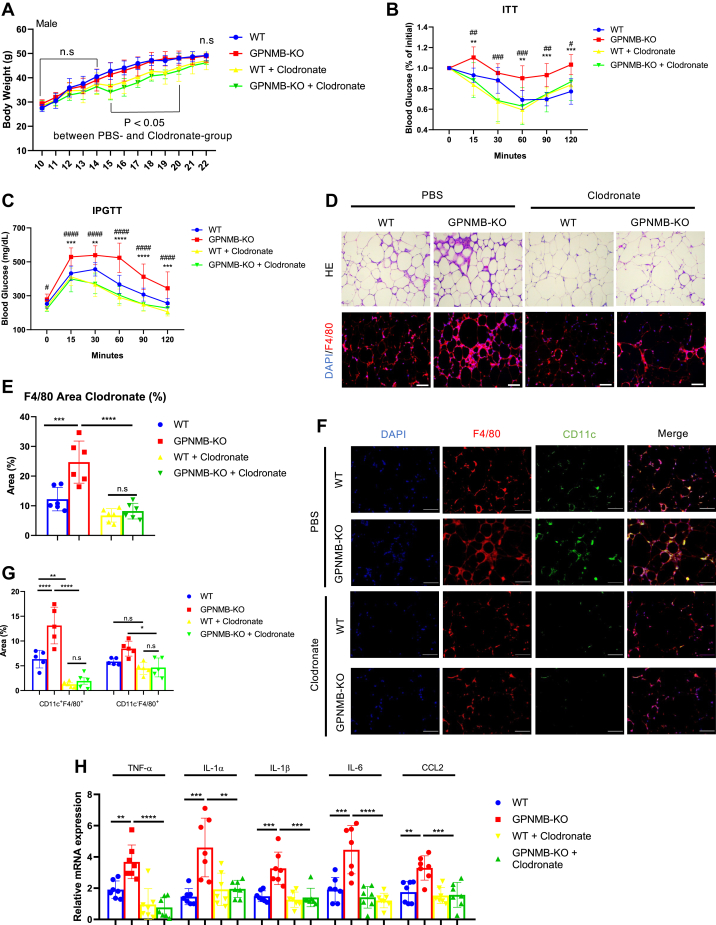
**Macrophage depletion abolishes the exacerbated metabolic disorders in male GPNMB-KO mice fed an HFD.***A*, body weight of male WT and GPNMB-KO mice fed an HFD with or without treatment with clodronate liposomes (n = 10 for WT; n = 9 for KO; n = 9 for WT + Clodronate; n = 7 for KO + Clodronate). The body weight was similar between WT and KO mice in the same treatment group. Clodronate-treatment group showed significantly less body weight than in PBS group during 5 to 10 weeks of the HFD feeding. *B*, ITT in male WT and GPNMB-KO mice fed an HFD for 12 weeks with or without treatment with clodronate liposomes (n = 6 for WT; n = 7 for KO; n = 5 for WT + Clodronate; n = 5 for KO + Clodronate). *C*, IPGTT in male WT and GPNMB-KO mice fed an HFD for 12 weeks with or without treatment with clodronate liposomes (n = 8 for WT; n = 9 for KO; n = 7 for WT + Clodronate; n = 7 for KO + Clodronate). *D*, representative images of H-E staining and immunohistochemistry for F4/80 in the WAT of male WT and GPNMB-KO mice fed an HFD for 14 weeks with or without treatment with clodronate liposomes. Bars: 100 μm. *E*, quantitative analysis for F4/80-positive area in the immunohistochemistry shown in (*D*) (n = 6 each). *F*, representative images of immunohistochemistry for CD11c and F4/80 in the WAT of male WT and GPNMB-KO mice fed an HFD for 14 weeks with or without treatment with clodronate liposomes. Bars: 50 μm. *G*, quantitative analysis for CD11c- and F4/80-double positive or CD11c-negative and F4/80-positive areas in the immunohistochemistry shown in (*F*) (n = 5 each). *H*, quantitative PCR for TNF-α, IL-1α, IL-1β, IL-6, and CCL2 in the WAT of male WT and GPNMB-KO mice fed an HFD for 14 weeks with or without treatment with clodronate liposomes (n = 7 each). Data represent mean ± SDM. ∗*p* < 0.05, ∗∗*p* < 0.01, ∗∗∗*p* < 0.001, and ∗∗∗∗*p* < 0.0001. ^#^*p* < 0.05, ^##^*p* < 0.01, ^###^*p* < 0.001, and ^####^*p* < 0.0001. In *B* and *C*, ∗ for comparison between WT and KO; ^#^ for comparison between KO + PBS and KO + Clodronate. One-way ANOVA with Tukey’s post hoc test for multiple comparisons was used for the analysis of the differences between each group (*E*, *G* and *H*), while two-way ANOVA with post hoc analysis of Fisher’s LSD for multiple comparison was used for the analysis of the differences between each group (*A*–*C*). n.s., not significant.

To elucidate a critical role of macrophages in the deteriorated WAT inflammation and the exacerbated obesity-related metabolic disorders in GPNMB-KO mice, we depleted macrophages in mice using clodronate liposomes. Clodronate liposomes were injected intraperitoneally once in 2 weeks during the HFD-feeding period (Fig. S7). Clodronate administration caused temporal body weight reduction in HFD-fed mice, but the body weight became similar 12 weeks after HFD feeding between the mice of vehicle and clodronate groups (Fig. 5*A*). Of note, exacerbated metabolic disorders in GPNMB-KO mice were abolished by administrating clodronate liposomes (Fig. 5, *B* and *C*). Histological analysis revealed the substantial reduction of macrophage infiltration in the WAT, especially in GPNMB-KO mice fed an HFD (Fig. 5, *D* and *E*). This reduction of ATMs was mostly because of the depletion of CD11c-positive recruited macrophages (Fig. 5, *F* and *G*). Consistently, deteriorated chronic inflammation in the WAT of GPNMB-KO mice was abrogated by the clodronate treatment (Fig. 5*H*). Hepatosteatosis and liver inflammation were also ameliorated in GPNMB-KO mice treated with clodronate (Fig. S8). These data sufficiently indicate that macrophage dysfunction is a primary cause for the exacerbated metabolic disorders in obese GPNMB-KO mice.

## Discussion

Chronic inflammation in the WAT is critically involved in obesity-related metabolic disorders. In this study, we identified that GPNMB is highly expressed in macrophages and hypertrophied adipocytes, and that it plays crucial role in the WAT inflammation during obesity (Fig. S9). It has been reported that GPNMB expression is increased in the WAT of obese mice and in the liver with reduced lipogenic capacity (10, 11). In one report, it has been shown that target activation of GPNMB in adipocytes ameliorated the fat accumulation and fibrosis of the liver in diet-induced obesity model without affecting the adiposity and glucose metabolism (11). In this previous study, DBA2J mice, in which missense mutation of GPNMB (R150X) occurs naturally, was also investigated for obesity-related metabolic disorders. DBA2J mice showed body weight, adiposity, glucose tolerance, and insulin sensitivity similar to those in WT mice fed with high-fat and high-sucrose diet. On the other hand, another previous report showed that GPNMB inhibition by a neutralizing antibody or liver-specific knockdown reduced body weight and improved obesity-related metabolic disorders (12). Therefore, the role of GPNMB in obesity and its-related metabolic disorders was controversial.

Our current study using the GPNMB-KO mice clearly showed that GPNMB plays a protective role against obesity-related metabolic disorders without affecting obesity and/or adiposity. The different results from the previous report using DBA2J mice were probably because of the incomplete knockout of GPNMB in DBA2J mice. The naturally occurring missense mutation of GPNMB caused no production of mature membrane-anchored GPNMB; however, truncated GPNMB extracellular domain can be produced and secreted in DBA2J mice. We show in this study that soluble secreted form of GPNMB-ECD rather than membrane-anchored full-length GPNMB plays a pivotal role in the inhibition of macrophage inflammability; therefore, truncated GPNMB-ECD might be partially functional and have some anti-inflammatory capacities in DBA2J mice. In addition, different diet may also have caused inconsistent results. For another report, experimental procedures such as administration of a neutral antibody and adeno-associated virus-expressing short hairpin RNA for GPNMB might cause unspecific off-target and/or unexpected biological effects, leading to the different results from ours.

In contrast to the exacerbated metabolic disorders in male obese GPNMB-KO mice, female GPNMB-KO mice showed no significant difference of insulin sensitivity and glucose tolerance from those in WT mice even after HFD feeding. These results suggest a gender-specific role of GPNMB in metabolic homeostasis; however, careful consideration is needed to interpret the data because female mice are generally resistant to diet-induced obesity comparing to male mice. Although HFD-fed female mice gain more weight than mice fed with normal chow, the weight gain is far little as compared with that in HFD-fed male mice. Deteriorated WAT inflammation associated with augmented macrophage infiltration is causally involved in the exacerbated metabolic disorders in male obese GPNMB-KO mice. Therefore, the protective role of GPNMB in the obesity-related metabolic disorders might be significant in considerably obese condition but not in mild obese condition. Notably, we regularly used peritoneal macrophages isolated from female mice for the *ex vivo* experiments; therefore, anti-inflammatory function of GPNMB in macrophages should be active in female mice. Further analysis is certainly required to elucidate a role of GPNMB in metabolic homeostasis in female mice.

CD44 is a transmembrane glycoprotein that has a wide variety of biological functions such as axion guidance, lymphocyte activation, cytokine signaling, and cell–cell adhesion; therefore, CD44 is involved in various diseases including arthritis, encephalitis, atherosclerosis, and obesity-related metabolic disorders (27). Consistent with the previous reports, we showed the GPNMB binding to CD44 and revealed that blocking the CD44 is critically involved in the GPNMB-mediated anti-inflammatory effects in macrophages. Our data showed that CD44 inhibition did not show significant anti-inflammatory effects in WT TEPMs, while it significantly ameliorated inflammatory capacity in GPNMB-KO TEPMs. These data suggest that endogenously expressed GPNMB sufficiently blocks the CD44-mediated accentuation of inflammatory signaling at least in our experimental settings. Genetic deletion as well as antibody-mediated inhibition of CD44 ameliorated obesity-related metabolic disorders in association with reduced WAT inflammation and hepatosteatosis despite the similar body weight in mice fed with HFD (28, 29). These metabolic phenotypes are exactly opposite to those in GPNMB-KO mice we described in this study and therefore further support a critical interaction of GPNMB with CD44 to elicit its beneficial anti-inflammatory functions to preserve metabolic homeostasis in obesity. However, detailed mechanisms by which GPNMB-binding affect the CD44 function remain unclear, and there might be other receptors/effectors for GPNMB to regulate macrophage inflammability besides CD44. Further investigation is required to elucidate a definite mode of action for GPNMB.

The limitation of our study is to use the null knockout mice. Therefore, differential contribution of adipocyte-derived and macrophage-derived GPNMB in the protection against obesity-related metabolic disorders remains unclear. Hepatocyte-derived GPNMB may also play some role. To elucidate the tissue- and/or cell-type specific role of GPNMB, further experiments using tissue-specific conditional knockout mice are required in the future. We clearly showed that macrophages are critically involved in the exacerbated obesity-related metabolic disorders in GPNMB-KO mice by macrophages depletion experiments. Bone marrow transplantation experiments will provide further information, especially for a role of bone-marrow-derived recruited macrophages; therefore, analysis of bone marrow chimeric mice is preferred to confirm the critical role of macrophages in the GPNMB-mediated protection against obesity-related metabolic disorders. We assessed the amount of recruited and resident ATMs using immunohistochemistry for CD11c and F4/80 in the WAT; however, flow cytometry is preferred for more precise and detailed analysis for ATMs subpopulation.

Our *in vitro* and *ex vivo* experiments suggested that GPNMB expressed in macrophages negatively regulates inflammatory capacities in an autocrine manner. It is also unclear whether exogenous supplementation of soluble secreted GPNMB by hypertrophied adipocytes could further reduce the inflammability in macrophages that express endogenous GPNMB. Considering the remarkable induction of GPNMB in adipocytes of obese mice, we presume that abundantly produced soluble GPNMB-ECD by hypertrophied adipocytes might play some roles in the inhibition of WAT inflammation during obesity, though further analyses are required to elucidate this issue. In this study, we revealed a protective role of GPNMB in obesity-related metabolic disorders largely through reducing macrophage inflammability, and thus GPNMB activation may provide a therapeutic strategy in obesity and diabetes. To further investigate the therapeutic potential of GPNMB in obesity-related metabolic disorders, beneficial effects of GPNMB-ECD supplementation in metabolic homeostasis in WT obese mice need to be explored in the future.

## Experimental procedures

### Materials

Antibody for GPNMB (#AF2330) was obtained from R&D systems. Antibody for CD44 (#553131) was obtained from BD Pharmingen. Antibody for NFκB p65 (#39370) was obtained from Active Motif. Antibodies for F4/80 (#ab6640) and Alexa Flour 488-labeled CD11c (#ab33503) were purchased from Abcam. Antibodies for phospho-NFκB p65 (#3033), phospho-Akt (#9271), total-Akt (#9272), and GAPDH (#2118) were purchased from Cell Signaling Technology. Antibody for FLAG (#F3165) was obtained from Sigma.

### Signal sequence trap

Signal sequence trap was performed as previously described (30, 31). Briefly, RNA was extracted from the WAT of obese mice fed with HFD for 14 weeks, followed by construction of cDNA library. After adding the BstXI adaptor, cDNAs underwent column chromatography, and cDNAs less than ∼500 bp were collected. These cDNAs were subcloned into pME-SST of retroviral backbone vector that contains the sequence encoding the constitutive active thrombopoietin receptor (MPL) downstream of the cloning site. These plasmids were transfected into Ba/F3 cells, which cannot survive in the absence of IL-3, *via* retroviruses. After infection, cells were plated into ∼1000 wells of 96-well plate in the absence of IL-3 and cultured for ∼1 week. Only cells transfected with cDNA that encodes signal sequence upstream of the constitutive active MPL can survive and proliferate in the absence of IL-3. Genomic DNAs of Ba/F3 cells that survive in the absence of IL-3 were extracted, and the subcloned cDNA was amplified by PCR, followed by direct sequencing.

### Cell culture

3T3-L1 preadipocytes were obtained from Health Science Research Resources Bank (#JCRB9014). Cells were cultured in DMEM supplemented with 10% FBS and 1% penicillin-streptomycin. Adipogenesis was induced to prepare differentiated 3T3-L1 adipocytes. Confluent 3T3-L1 preadipocytes were treated with 0.25 μM dexamethasone, 0.2 μM insulin, and 0.5 mM isobutylmethylxanthine at day 0 for 48 h, followed by another 48 h incubation with 0.2 μM insulin. Subsequently, cells were cultured in DMEM supplemented with 10% FBS and 1% penicillin-streptomycin. 3T3-L1 adipocytes at 10 to 12 days postdifferentiation were regularly used for experiments. Lentiviruses carrying GFP or GPNMB were generated by transfecting target genes in pLenti vector, psPAX2 and pMD2.G plasmids into HEK293 cells. 3T3-L1 adipocytes were incubated with lentivirus carrying either GPNMB or GFP for 24 h, followed by incubation with fresh growth medium for 96 h before using for experiments. Sufficient transfection efficacy was confirmed by detecting GFP signals more than 90% cells.

RAW264.7 cells were regularly cultured in DMEM supplemented with 10% FBS and 1% penicillin-streptomycin. Short interfering (si)RNAs for silencing GPNMB was performed using siGENOME SMARTpool Mouse Gpnmb (Dharmacon) or Silencer Negative Control siRNA (Thermo Fisher Scientific) for the control group. Lipofectamine RNAiMax (Thermo Fisher Scientific) was used to deliver siRNA according to the manufacturer’s instructions. In some experiments, macrophages were treated with recombinant soluble GPNMB-ECD (Lys23-Asn486: R&D Systems #2550-AC) (1 μg/ml) or anti-CD44 antibody (1:200), 48 h after siRNA transfection. Lipopolysaccharide (LPS) (10 ng/ml) treatment was also performed 48 h after siRNA transfection.

### Animal study

All experimental protocols were approved by the Ethics Review Committee for Animal Experiments of Kobe Pharmaceutical University (#2019-045). All researchers have complied with all relevant ethical regulations. GPNMB-KO mice (C57BL6N background) were generated using cryo-preserved mouse sperm obtained from KOMP (Gpnmb^tm1(KOMP)Vlcg^).

Mice were housed in specified cages of adequate size (1–3 mice per cage) in an animal facility with properly controlled temperature (23 °C) and humidity (60%). Mice were maintained under 12-h light/12-h dark cycle and fed either normal chow (NC) diet (containing 5.1% fat and 23.1% protein) or HFD (Oriental Bio HFD-60) (containing 35% fat, 23% protein, and 25.3% carbohydrates) with *ad libitum* access to water and food. For the HFD feeding, 10-week-old male mice were maintained on a HFD for up to 14 weeks. Insulin and glucose tolerance tests (ITT and ipGTT) were performed as previously described. For ITT, mice were administered 1 or 2 IU/kg human insulin by subcutaneous injection without fasting. For ipGTT, mice fasted for 6 h and 0.75 or 1.5 g/kg D-glucose were intraperitoneally injected. Glucose oxidase method (Johnson & Johnson K. K.) was used to measure blood glucose level. Visceral perigonadal WAT and liver were used for all analysis.

### Isolation of SVF and mature adipocytes

WATs harvested from mice were minced in 0.15% collagenase type I (Worthington) in Hank’s Balanced Salt Solution (HBSS) (Gibco). The homogenate was placed for 40 to 60 min in 37 °C shaking water bath. The tissue digest was centrifuged at 1000 rpm for 10 min to separate the floating mature adipocytes from SVF pellet.

### Isolation of endothelial cells and macrophages by magnetic-activated cell sorting

SVF was subjected to magnetic sorting using anti-CD11b magnetic microbeads (Miltenyi) for macrophage isolation. Afterward, the cells in the flow-through were subjected to magnetic sorting using anti-CD31 magnetic microbeads (Miltenyi) for endothelial cells isolation. The remaining cells in the flow-through after endothelial cells isolation were used as others in experiments.

### Macrophage depletion using clodronate liposomes

The mice were administered with either clodronate liposomes (Hygieia Bioscience) (6.3 mg/kg) or PBS intraperitoneally during the HFD feeding (Fig. S7). Injection of clodronate or PBS was started at week 2 of the HFD feeding, and the injection was performed every 2 weeks until week 10. Metabolic analyses of these mice were performed at week 12.

### Peritoneal macrophage isolation

TEPMs were isolated as follows. Briefly, 4% thioglycollate solution (Fluka) in 1 ml PBS was administered intraperitoneally to 8- to 10-week-old female WT and GPNMB-KO mice. After 5 days, the peritoneal cavity was injected with sterile cold PBS, followed by fluid collection. To collect resident peritoneal macrophages, lavage fluid was collected using female mice without thioglycolate injection. The fluid was centrifuged at 3000 rpm for 5 min at 4 °C, and the collected cells were resuspended using RPMI 1640 medium (Thermo Fisher Scientific) containing 10% fetal bovine serum (FBS) (Biowest) and 1% penicillin-streptomycin (Thermo Fisher Scientific). Nonadherent cells were removed by washing with prewarmed RPMI 1640 medium after 2 h of incubation to enable adherence of macrophages. PMs were regularly isolated from female WT and GPNMB-KO mice for experiments.

TNF-α (10 ng/ml) treatment was performed after 24 h of macrophage isolation. In some experiments, macrophages were treated with recombinant soluble GPNMB-ECD (R&D Systems) (1 μg/ml) or anti-CD44 antibody (1:200), which was performed after 24 h of macrophage isolation. To prepare the conditioned medium (CM), macrophages were given serum-free RPMI 1640 medium, followed by collection of supernatants after 24 h of incubation.

### Histological staining

WAT and liver tissues were fixed with 4% paraformaldehyde (PFA) (Wako Pure Chemical) for 24 h prior to dehydration and paraffin embedding, followed by cutting into 3 μm sections for liver and 5 μm sections for WAT. Those sections were stained with hematoxylin and eosin to evaluate their structural differences. Keyence BZ-X800 microscope (Keyence) was used to capture images of those sections.

### Immunofluorescence staining

After deparaffinization, 10 mmol/l boiled citrate buffer (pH 6.0) as antigen unmasking solution (Vector Laboratories) was applied for 15 min to the sections. After cooling at room temperature for approximately 30 min, the sections were washed with PBS, blocked with 5% donkey serum in PBS-T, followed by incubation with anti-F4/80 (Abcam) and Alexa488-labeled anti-CD11c (Abcam) antibodies at 4 °C for overnight. Afterward, sections were washed with PBS-T and then incubated with Alexa Flour 594-labeled anti-rat secondary antibody (Thermo). Following the washing, sections were covered with VECTASHILED mounting medium with DAPI (Vector Laboratories). Sections were observed under fluorescence microscopy (Keyence BZ-X800).

### Quantitative real-time PCR

RNAs were extracted from tissues or cells except the WAT and 3T3-L1 adipocytes using RNAiso Plus (TAKARA), followed by purification using NucleoSpin RNA Clean-Up kit (Macherey-Nagel). RNAs of WAT and 3T3-L1 adipocytes were extracted using QIAzol lysis reagent (QIAGEN), followed by purification using RNeasy Lipid Tissue Mini Kit (QIAGEN). PrimeScript RT Reagent Kit with gDNA eraser (TAKARA) was used for cDNA synthesis from 1 μg of total RNA. Amount of RNA was always identical among samples. The cDNA synthesis protocol included a DNase digestion step. The LightCycler96 (Roche Science) was used to conduct quantitative real-time PCR with FastStart SYBR Green Master (Roche Applied Science). The mRNA expression levels of the target genes were normalized relative to 18S levels and the data were presented in arbitrary units.

### Immunoblotting

RIPA buffer containing protease and phosphatase inhibitors was used to lyse cell or tissues. The concentration of protein samples was equalized before boiling in sample buffer. Subsequently, samples were separated by SDS-PAGE, followed by transferring onto nitrocellulose membrane. Membranes were blocked with 5% skim milk in TBS-T for 30 min prior to probing with first antibody diluted in blocking buffer for appropriate duration for overnight at 4 °C, followed by incubation in secondary antibody diluted in blocking buffer. The signals were visualized using Amersham ECL (GE Healthcare) and detected using ChemiDoc XRS+ (BioRad). Signals were quantified and normalized with GAPDH expression levels with the data presented in arbitrary units.

### Immunoprecipitation

Expression constructs for FLAG-tagged GPNMB-ECD were transfected into HEK293 cells using lipofectamine 3000 (Thermo). Fresh growth medium was given 24 h after transfection and further incubated for 24 h. Cells were then incubated with serum free medium for another 24 h, and subsequently conditioned medium containing secreted GPNMB-ECD was collected. Transfection of empty vector was performed to prepare the control conditioned medium (MOCK). RAW264.7 macrophages were incubated with the conditioned medium for 1 h. Those macrophages were lysed with CelLytic M lysis buffer (Sigma) followed by precleaning with IgG-agarose (Sigma). Subsequently, cell lysates were incubated with anti-FLAG M2 antibody (Sigma) or normal rabbit IgG (Santa Cruz) at 4 °C for overnight, followed by incubation with protein G-agarose (Thermo) at 4 °C for 2 h. Immunoprecipitated protein was released by boiling in protein sample buffer, followed by SDS-PAGE. Coprecipitation of CD44 was detected by immunoblotting using anti-CD44 antibody (Abcam).

### Statistical analysis

All data are presented as mean ± standard deviation of the mean (SDM). Two-tailed Student’s *t* test was used to examine differences between groups. Comparisons among more than three groups were assessed for significance by one-way or two-way ANOVA with Tukey’s post hoc test or Fisher’s exact test. *p* < 0.05 was considered statistically significant. All statistical analysis was performed using GraphPad Prism 8 (GraphPad Software Inc).

## Data availability

All data are contained within the manuscript.

## Supporting information

This article contains supporting information (Figs. S1–S9, Supplementary Data 1).

## Conflict of interest

The authors declare that they have no conflicts of interest with the contents of this article.
