# Supplementary material for: GPNMB plays a protective role against obesity-related metabolic disorders by reducing macrophage inflammatory capacity
Source: J Biol Chem. 2021 Sep 25;297(5):101232. doi: 10.1016/j.jbc.2021.101232 (PMC8524194; doi:10.1016/j.jbc.2021.101232)
Supplement: Supplemental Figures S1–S9 [file mmc1.pdf]

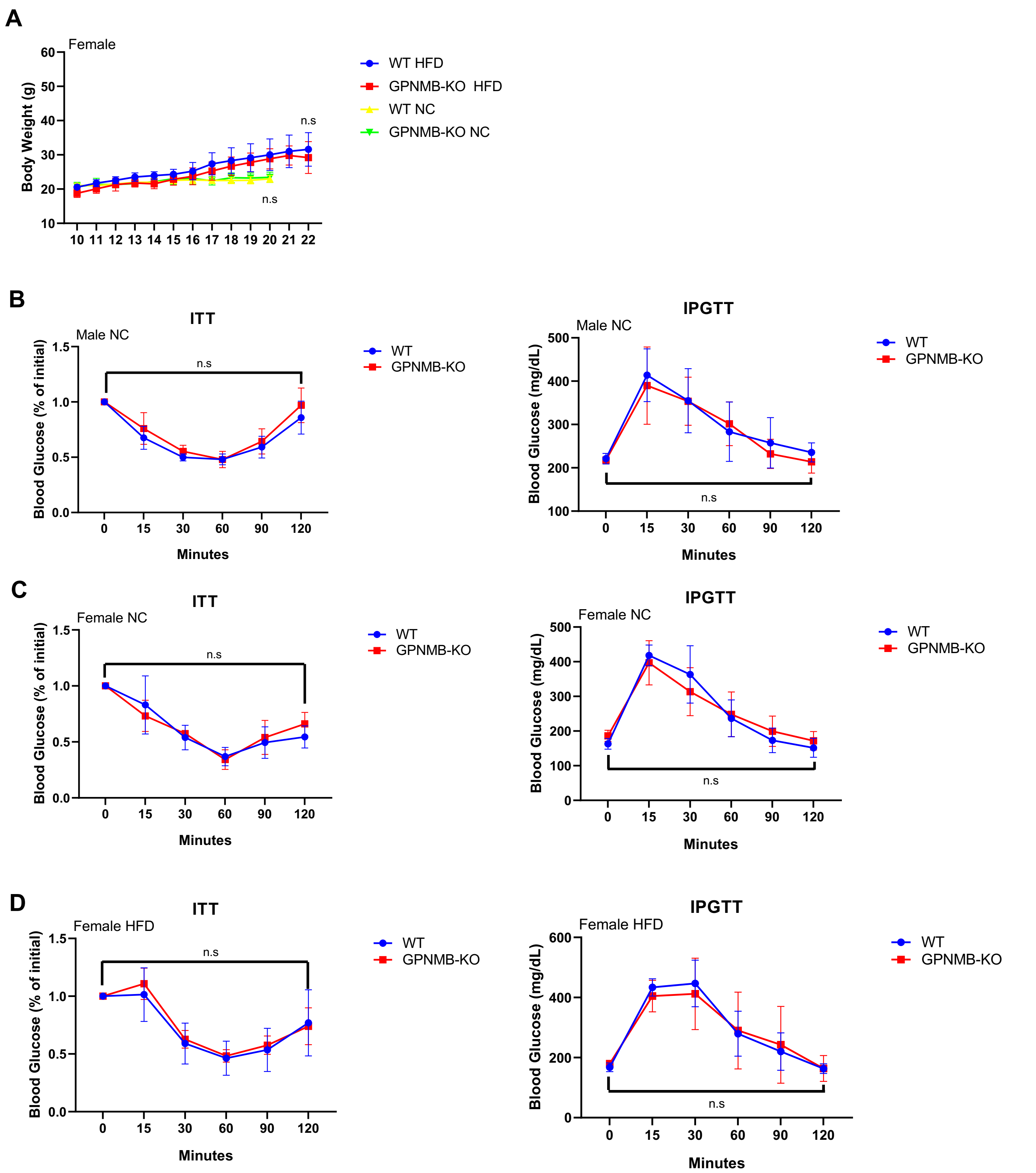

Supplementary Fig 1. Metabolic phenotype in male mice fed with NC and in female mice fed with NC or HFD. **(A)** Body weight of female WT and GPNMB-KO mice fed either normal chow (NC) or HFD at the indicated ages of weeks (n = 6 for WT HFD; n = 5 for GPNMB-KO HFD; n = 7 for WT NC; n = 6 for WT HFD). **(B)** ITT and IPGTT in male WT and GPNMB-KO mice fed NC for 10 weeks (n = 5 each). **(C)** ITT and IPGTT in female WT and GPNMB-KO mice fed NC for 10 weeks (n = 6 for WT; n = 5 for KO). **(D)** ITT and IPGTT in female WT and GPNMB-KO mice fed HFD for 12 weeks (n = 5 each). Data represent mean ± SDM. Two-tailed student's *t*-test was used for the analysis of the differences between groups (A-D).

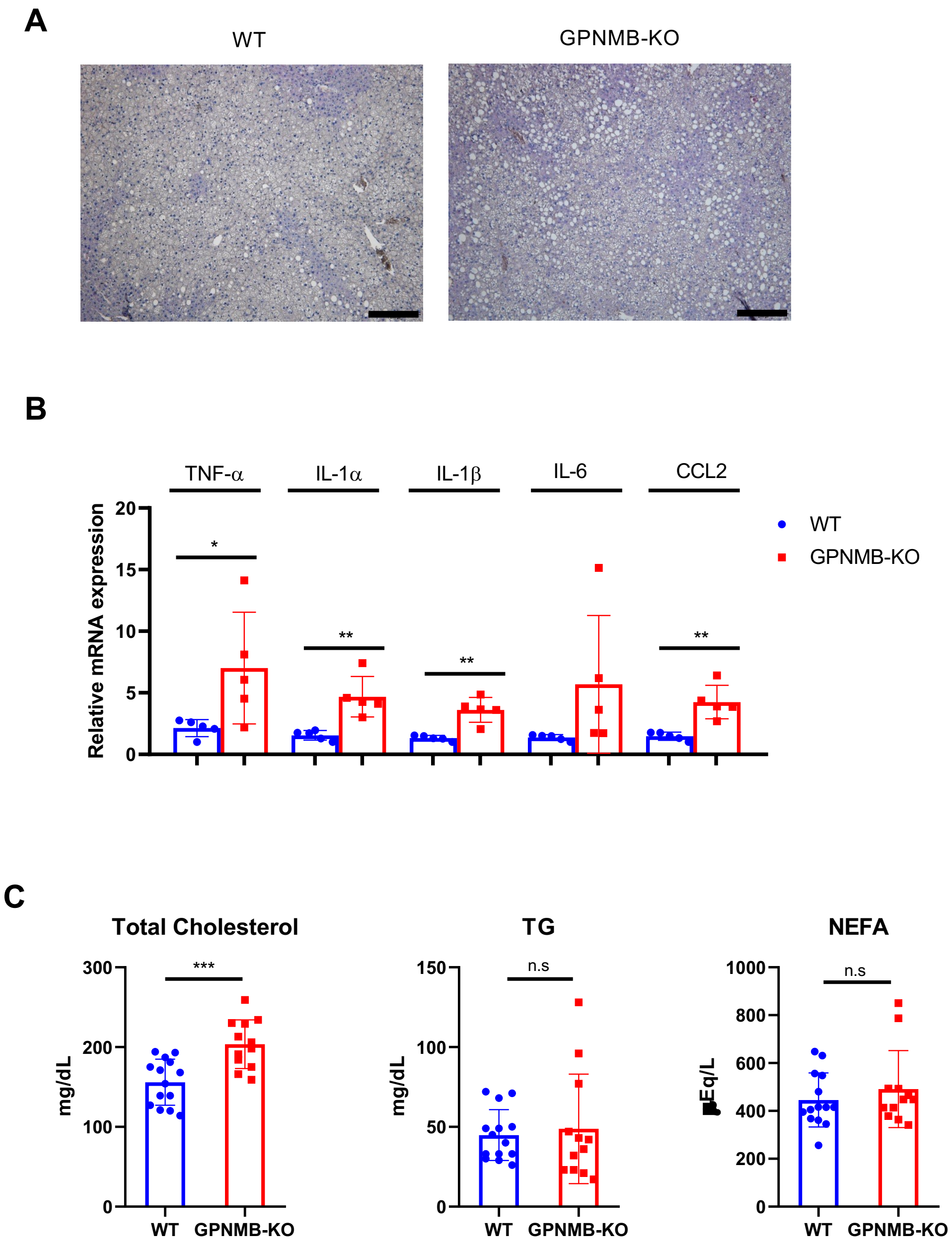

Supplementary Figure 2. Liver phenotype and serum lipid profiles in HFD-fed male WT and GPNMB-KO mice. **(A)** Representative images of hematoxylin and eosin staining of liver sections prepared from male WT and GPNMB-KO mice fed with HFD. Bar = 200 μm. **(B)** Quantitative PCR analysis for TNF-α, IL-1α, IL-1β, IL-6, and CCL-2 in liver isolated from male WT and GPNMB-KO mice fed with HFD (n = 5 each). **(C)** Serum lipid profiles in male WT and GPNMB-KO mice fed with HFD (n = 14 for WT; n = 12 for KO). Data represent mean $\pm$ SDM. *$^{*}P < 0.05$, $^{**}P < 0.01$, and $^{***}P < 0.001$.* Two-tailed student's *t*-test was used for the analysis of the differences between groups (B and C).

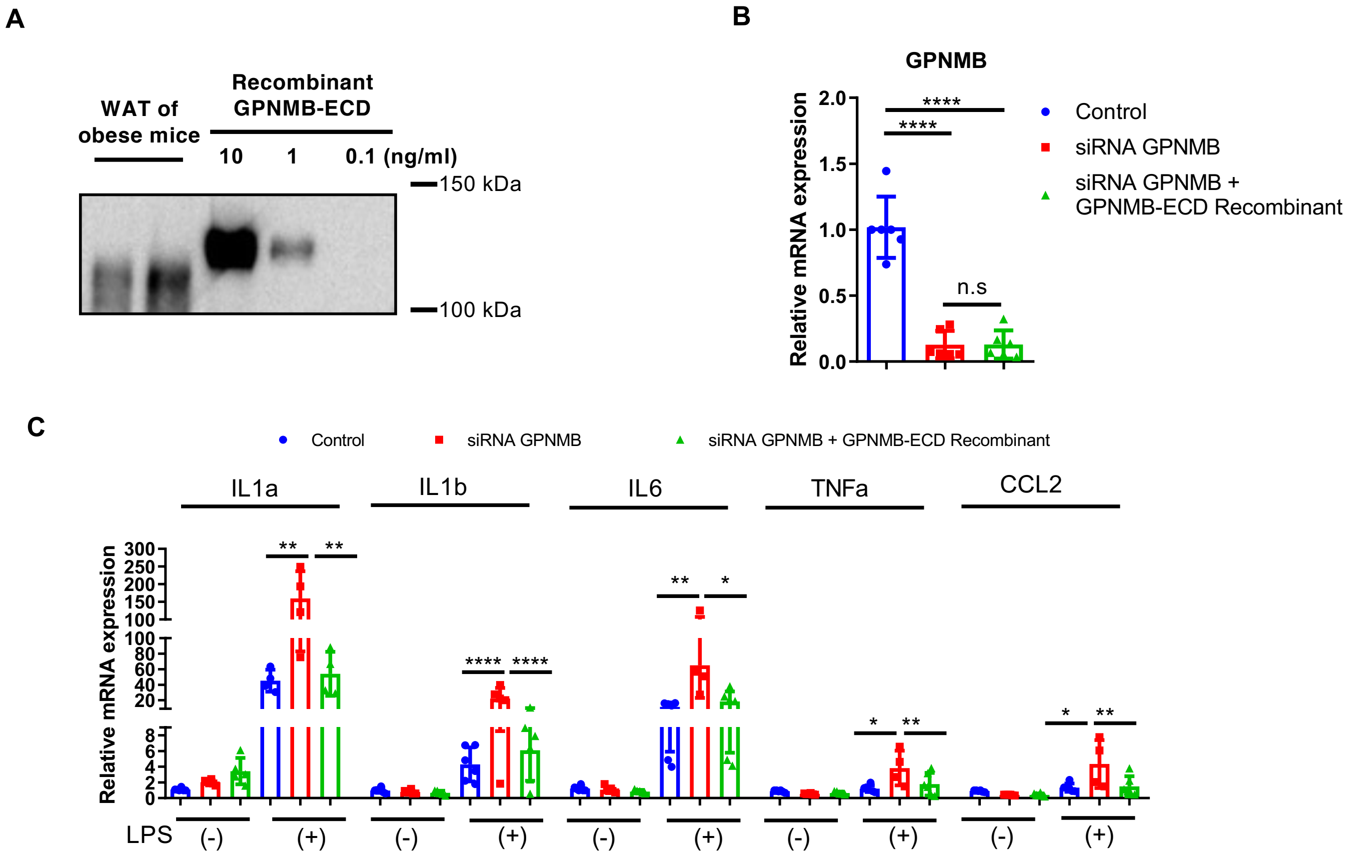

Supplementary Figure 3. Anti-inflammatory role of GPNMB in RAW264.7 macrophages.
(A) Immunoblotting for GPNMB in the WAT of obese mice (~10 mg/ml) and the recombinant GPNMB-ECD at various concentration. **(B)** Quantitative PCR analysis for GPNMB in RAW264.7 macrophages transfected with either negative (control) or GPNMB siRNA. Some cells transfected with GPNMB were cultured in the presence of recombinant GPNMB-ECD (n = 6 each). **(C)** Quantitative PCR analysis for TNF-α, IL-1α, IL-1β, IL-6, and CCL-2 in RAW264.7 cells with or without LPS-treatment. Cells were transfected with either negative (control) or GPNMB siRNA. Some cells transfected with GPNMB were cultured in the presence of recombinant GPNMB-ECD (n = 4-6 each). Data represent mean ± SDM. *$P < 0.05$, **$P < 0.01$, and ****$P < 0.0001$. One-way ANOVA with Tukey's post hoc test for multiple comparisons was used for the analysis of the differences between each group (B and C).

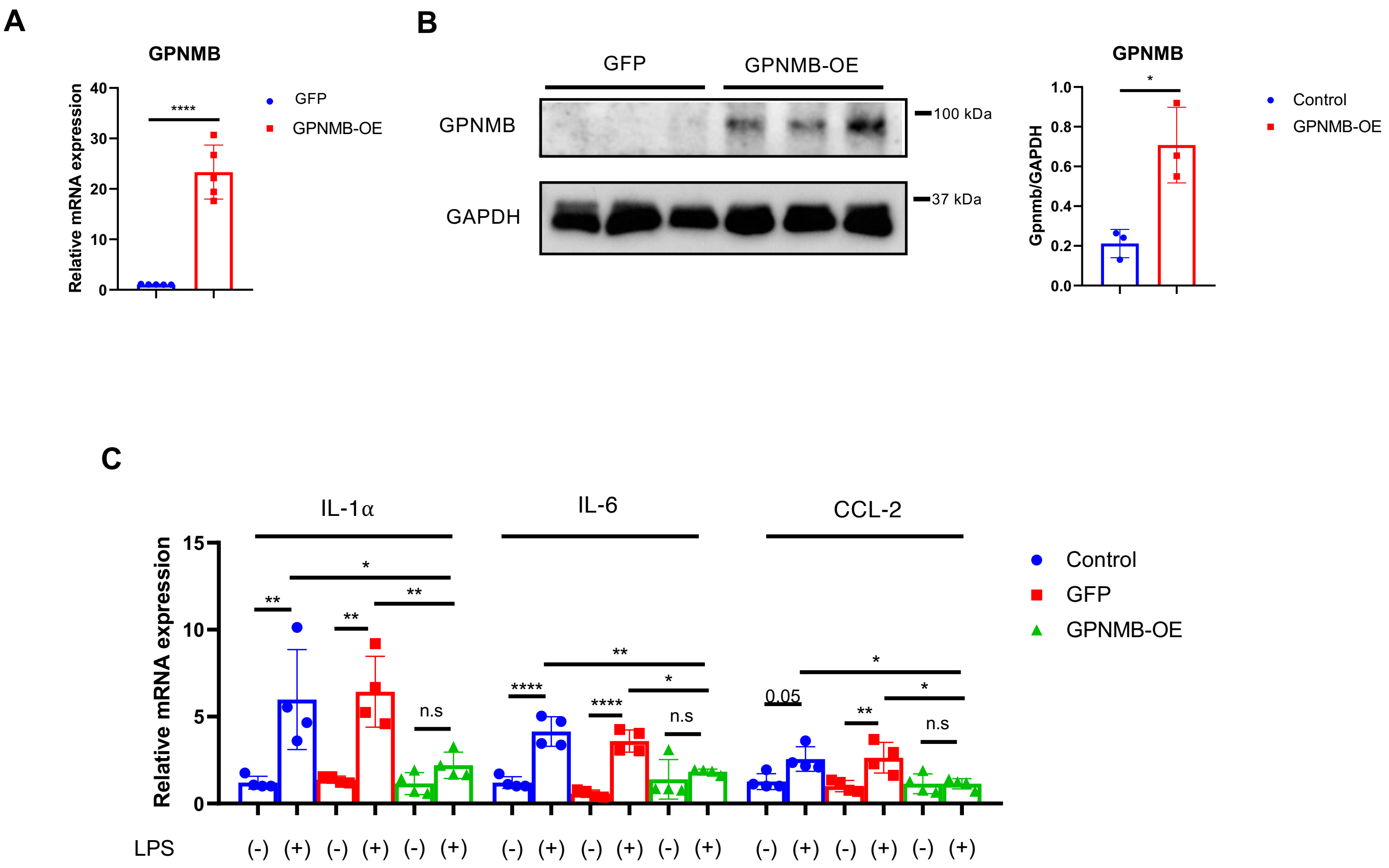

Supplementary Fig. 4. Adipocyte-derived GPNMB reduces macrophage inflammability.
(A) Quantitative PCR analysis for GPNMB in 3T3L1 adipocytes transfected with either GFP or GPNMB (GPNMB-OE) using lentiviruses (n = 5 each). **(B)** Immunoblotting for GPNMB in 3T3L1 adipocytes transfected with either GFP or GPNMB (GPNMB-OE) using lentiviruses (n = 3 each). **(C)** Conditioned medium was collected from 3T3-L1 adipocytes transfected with either GFP or GPNMB (GPNMB-OE), and then given to RAW264.7 macrophages. Some cells were given medium incubated without cells (control). Subsequently, cells were treated with LPS, and IL-1α, IL-6, and CCL-2 expressions were quantitatively analyzed (n = 4 each). Data represent mean $\pm$ SDM. *$^*P < 0.05$, $^{**}P < 0.01$, $^{***}P < 0.001$, and $^{****}P < 0.0001$.* Data are presented as mean $\pm$ SD. Two-tailed student's t-test was used for the analysis of the differences between two groups (A and B), while one-way ANOVA with Tukey's post hoc test for multiple comparison was used for the analysis of the differences between each group (C)**.**

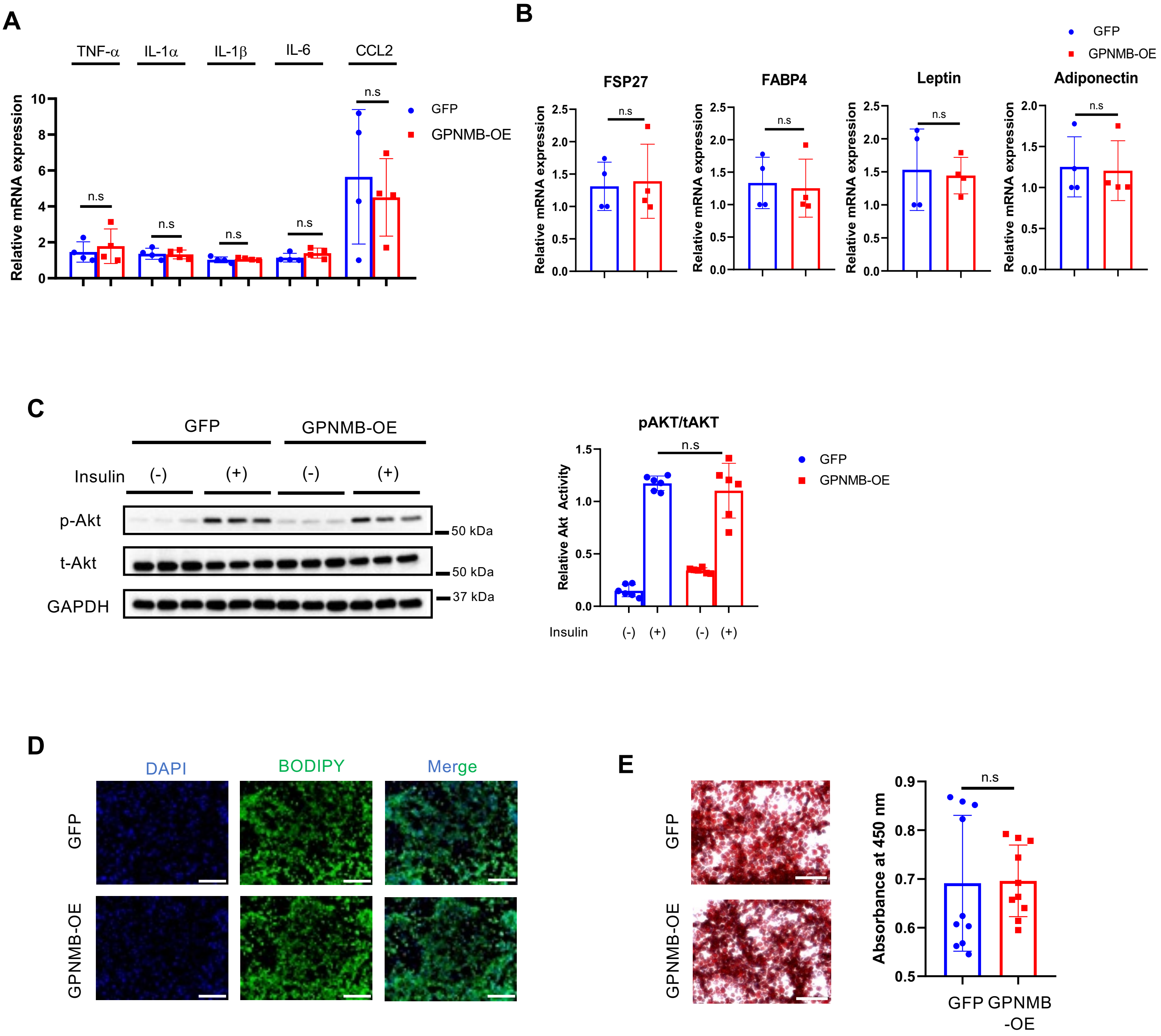

Supplementary Fig. 5. Minimal role of GPNMB in adipocyte biology.
(A) Quantitative PCR analysis for TNF-α, IL-1α, IL-1β, IL-6, and CCL-2 in 3T3L1 adipocytes transfected with either GFP or GPNMB (GPNMB-OE) using lentiviruses (n = 4 each). **(B)** Quantitative PCR analysis for FSP27, FABP4, Leptin, and Adiponectin expression in 3T3L1 adipocytes transfected with either GFP or GPNMB (GPNMB-OE) using lentiviruses (n = 4 each). **(C)** Immunoblotting for insulin signaling in 3T3L1 adipocytes transfected with either GFP or GPNMB (GPNMB-OE) using lentiviruses. Insulin-mediated Akt activation was quantified (n = 6 each). **(D)** Representative images of BODIPY-staining in 3T3L1 adipocytes transfected with either GFP or GPNMB (GPNMB-OE) using lentiviruses. Bars = 200 μm. **(E)** Representative images of ORO-staining in 3T3-L1 adipocytes transfected with either GFP or GPNMB (GPNMB-OE) using lentiviruses. Bars = 100 μm (left). ORO absorbance at 450 nm was quantified (n = 10 each; right). Data represent mean $\pm$ SDM. Two-tailed student's t-test was used for the analysis of the differences between two groups (A, B and E), while one-way ANOVA with Tukey's post hoc test for multiple comparison was used for the analysis of the differences between each group (C).

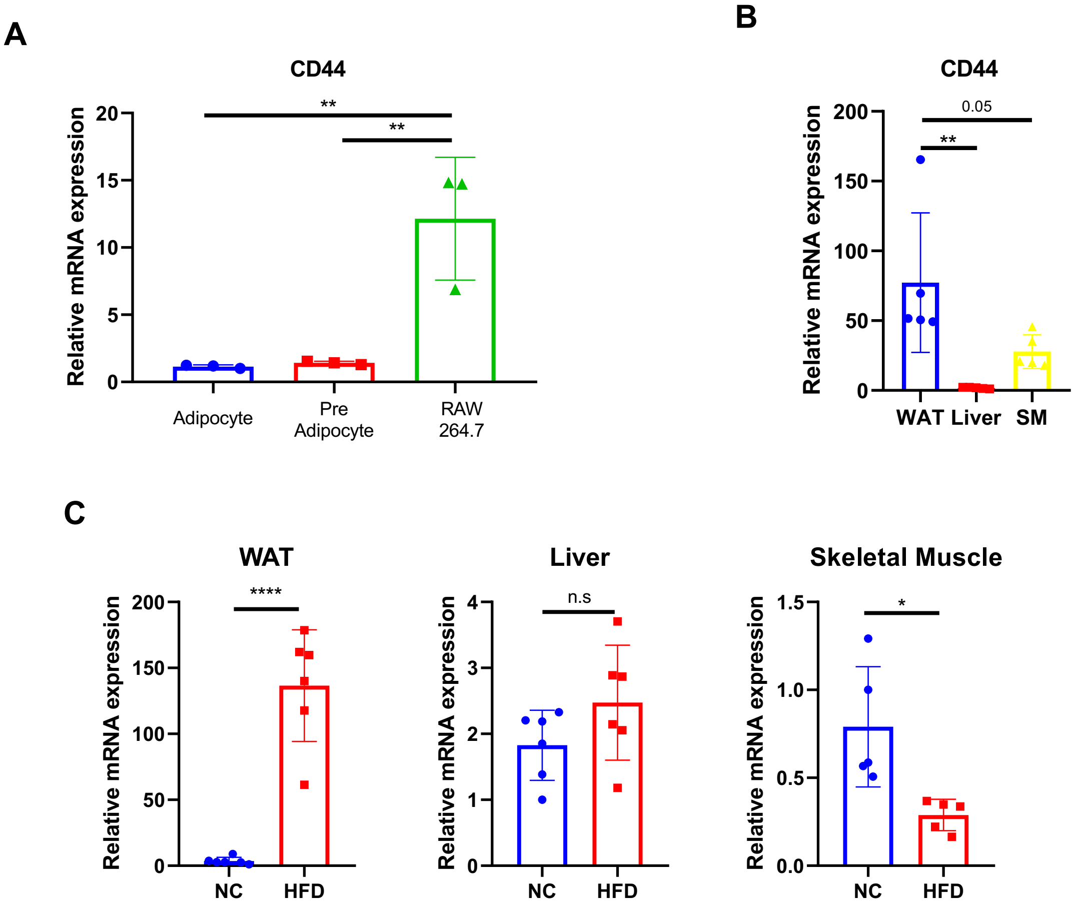

Supplementary Figure 6. CD44 is highly expressed in macrophages and in the WAT.
(A) Quantitative PCR analysis for CD44 in 3T3-L1 preadipocytes, 3T3-L1-derived adipocytes and RAW264.7 macrophages (n = 3 each). **(B)** Quantitative PCR for CD44 expression in the WAT, liver, and skeletal muscle (SM) isolated from male WT mice fed with normal chow (NC) (n = 5 each). **(C)** Quantitative PCR analysis for CD44 in the WAT, liver, and skeletal muscle isolated from male mice fed with NC or HFD (n = 6 for WAT and liver; n = 5 for skeletal muscle). Data represent mean ± SDM. **P < 0.05 and ****P < 0.0001.* Two-tailed student's t-test was used for the analysis of the differences between two groups (C), while one-way ANOVA with Tukey's post hoc test for multiple comparison was used for the analysis of the differences between each group (A and B).

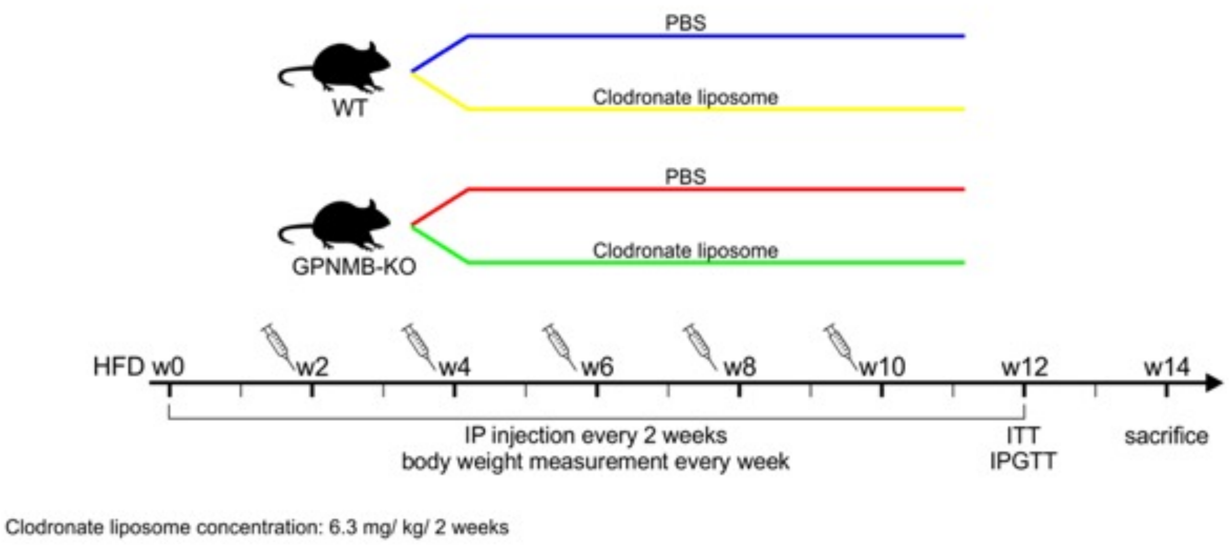

Supplementary Figure 7. Schematic diagram of clodronate experiments.

A

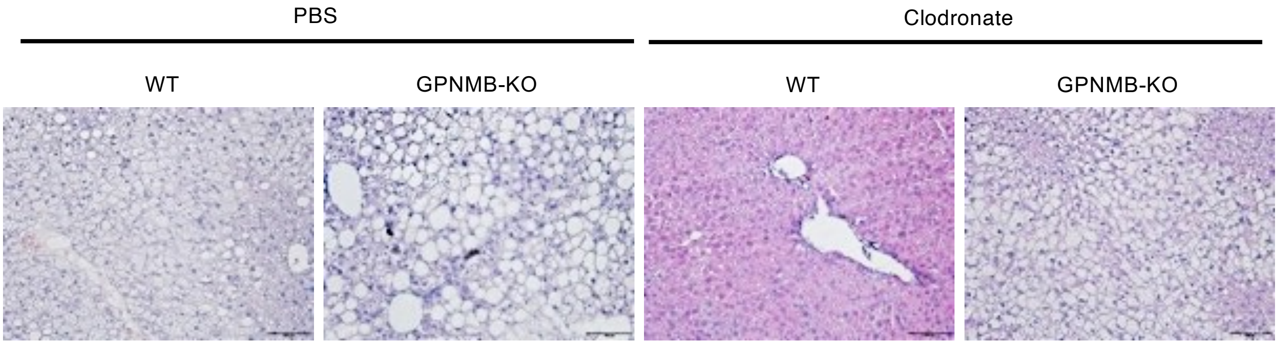

B

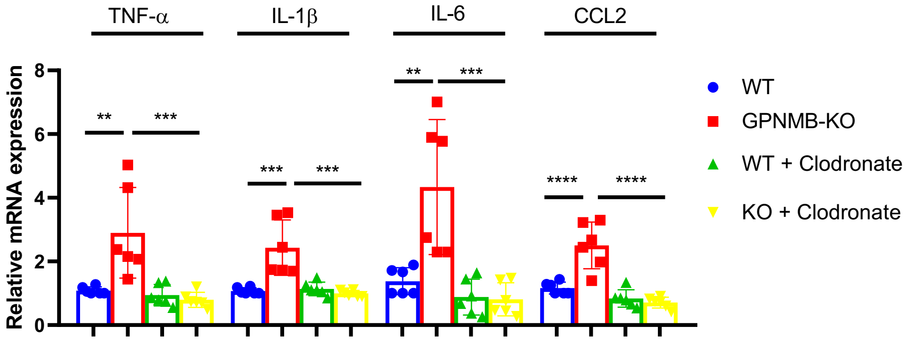

Supplementary Figure 8. Liver phenotypes in male HFD-fed WT and GPNMB-KO mice treated with clodronate liposomes.

(A) Representative images of H-E staining of liver sections prepared from male WT and GPNMB-KO mice fed with HFD for 14 weeks. Mice were injected with either PBS or clodronate liposomes once in 2 weeks during the HFD-feeding. Bar = 100 μm. **(B)** Quantitative PCR analysis for TNF-α, IL-1β, IL-6, and CCL-2 in liver isolated from male WT and GPNMB-KO mice fed an HFD for 14 weeks (n = 6 each). Mice were injected with either PBS or clodronate liposomes once in 2 weeks during the HFD-feeding. Data represent mean $\pm$ SDM. ***P < 0.01, ***P < 0.001, and ****P < 0.0001*. One-way ANOVA with Tukey's post hoc test for multiple comparison was used for the analysis of the differences between each group (B).

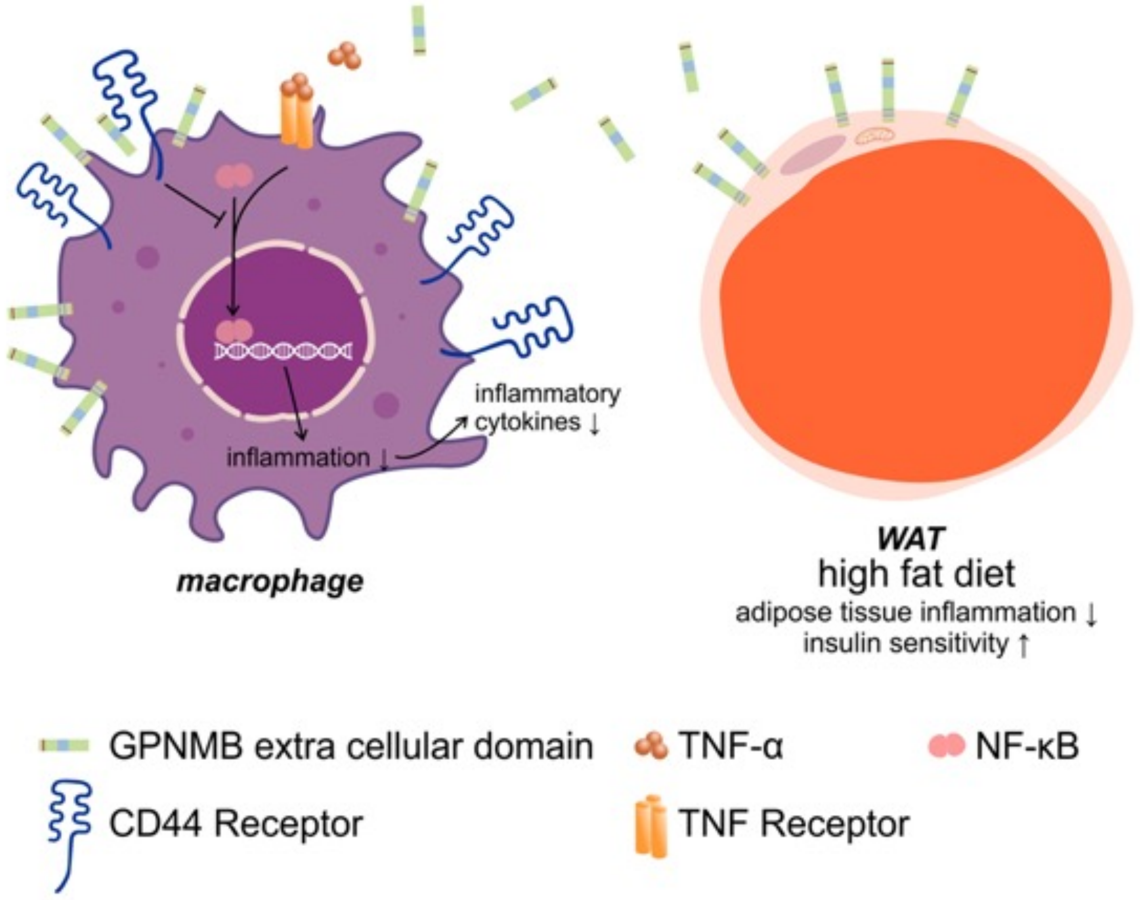

Supplementary Figure 9. Schematic diagram of the working model for a role of GPNMB in obesity-related metabolic disorders.

GPNMB is highly expressed in macrophages, and its soluble secreted ECD reduces their inflammatory capacities in an autocrine manner. GPNMB-ECD binds to CD44, leading to inhibition of CD44-mediated accentuation of NF-kB signaling. In obesity, GPNMB expression is remarkably upregulated in adipocytes, and adipocyte-derived GPNMB may also inhibit the macrophage inflammability in a paracrine matter in the WAT.
